# Elevated lipoprotein (a) and large apo(a) isoforms in Omani patients: a distinct cardiovascular risk profile

**DOI:** 10.1016/j.ajpc.2026.101781

**Published:** 2026-08-03

**Authors:** Amani Al Khaifi, Khalid Al Waili, Balaqis Al Shaibani, Hilal Al Sabti, Adil Al Riyami, Manal Al-Kindi, Fahad Zadjali, Khalid Al-Saidi, Fahad Al Kindi, Hatim Al Lawati, Khamis Al Hashmi, Abrar Al Zadjali, Razan Al Zadjali, Sana Al Hasari, Romuald Techer, Kevin Chemello, Mirdavron Mukaddirov, Koukab Al Farsi, Hassan Al Riyami, Suaad Al Mukhaini, Hala Al Badi, Mohsin Al-Saleh, Eman R. Abdelmonaem, Ibrahim Al-Zakwani, Gilles C. Lambert, Khalid Al-Rasadi

**Affiliations:** a Department of Biochemistry, College of Medicine and Health Sciences, Sultan Qaboos University, Muscat, Oman; b Medical Research Center, Sultan Qaboos University, Muscat, Oman; c Department of Clinical Biochemistry, Sultan Qaboos University Hospital, University Medical City, Muscat, Oman; d Ministry of Health, Muscat, Oman; e Department of Surgery, Sultan Qaboos University Hospital, University Medical City, Muscat, Oman; f Department of Medicine, Sultan Qaboos University Hospital, University Medical City, Muscat, Oman; g Department of Chemical Pathology, Royal Hospital, Ministry of Health, Muscat, Oman; h Dean office, Oman College of Health Sciences, Muscat, Oman; i Department of Cardiology, National Heart Center, Royal Hospital, Ministry of Health, Muscat, Oman; j Department of Physiology, College of Medicine and Health Sciences, Sultan Qaboos University, Muscat, Oman; k Université de La Réunion, Inserm, UMR1188 Diabète Athérothrombose Thérapies Réunion Océan Indien (DéTROI), Saint-Pierre, France; l School of Biomedical Sciences, Faculty of Medicine and Health, University of New South Wales, Sydney, NSW, Australia; m Department of Nursing, Sultan Qaboos University Hospital, University Medical City, Muscat, Oman; n Department of Biochemistry, Faculty of Pharmacy, Mansoura University, Mansoura, Egypt; o Department of Pharmacology & Clinical Pharmacy, College of Medicine and Health Sciences, Sultan Qaboos University, Muscat, Oman; p Department of Pharmacy, Sultan Qaboos University Hospital, University Medical City, Muscat, Oman

**Keywords:** Lipoprotein(a), Apolipoprotein(a) isoform size, Cardiovascular-disease, Familial hypercholesterolemia, Premature coronary artery disease, Middle Eastern population

## 1 Introduction

Elevated Lipoprotein(a) [Lp(a)] is the most common genetically inherited risk factor for cardiovascular diseases (CVD) and calcified aortic valve stenosis. Its signature protein, apolipoprotein (a) [apo(a)], contains a hypervariable number of kringle IV (KIV) domains accounting for up to 60% of Lp(a) variance [1]. Marked differences in Lp(a) concentrations and apo(a) size polymorphisms exist between ethnic groups, but data from Arab populations, who present with a higher CVD risk, remain scarce [2]. Investigating Lp(a) levels and apo(a) size polymorphism is needed to assess the specific Lp(a)-associated CVD risk, to establish adequate therapeutic thresholds, and to develop optimal clinical management strategies for populations from the Gulf region. We addressed this critical knowledge gap by measuring Lp(a) concentrations and assessing apo(a) isoform size in the Omani population.

## 2 Methods

### 2.1 Study cohort

596 Omani nationals were recruited from Muscat major hospitals including 174 healthy volunteers with no history of CVD, no cardiometabolic risk factor, no lipid-lowering treatment, and 422 patients with either familial hypercholesterolemia (FH), premature coronary artery disease (PCAD), or aortic valve stenosis (AVS). Demographic, biochemical, and clinical data (age, sex, BMI, smoking status, diabetes mellitus, hypertension, and medical history of CVD) were documented. The FH subgroup included patients with either probable or definite FH according to the Dutch Lipid Clinic Network criteria. The PCAD subgroup included patients who had a first incident acute coronary event confirmed by coronary angiography before 55/65 years of age in men/women. The AVS group included patients who underwent transcatheter aortic valve implantation due to severe degenerative aortic valve stenosis. The study was approved by the research ethics committees of Sultan Qaboos University and the Ministry of Health of Oman (REF. NO. SQU-EC/386/2021, MREC #2448).

### 2.2 Biochemistry

Plasma lipids were measured on a Roche COBAS C 501 analyzer. Plasma Lp(a) was measured using the apo(a) isoform-insensitive Tina-quant Lp(a) Gen. 2 immunoturbidimetric assay (Roche Diagnostics, Cat 5852641190) and categorized according to the EAS consensus statement [1]. Individual apo(a) isoform sizes were determined by Western Blot, using a validated protocol described elsewhere [3].

### 2.3 Statistical analysis

Statistical analyses were performed using StataNow 19.0 SE. Variables are presented as mean ± standard deviation or median and interquartile range (IQR). Comparisons between groups were made using the Kruskal–Wallis test, and correlations between variables using the Spearman’s rank test. The association between Lp(a) and CVD was assessed per 1nmol/L increase in Lp(a), and at the clinical cutoff values of 75 and 125nmol/L, using multivariable logistic regression models adjusted for sex, age, BMI, diabetes mellitus, hypertension, smoking, LDL, and HDL-cholesterol. Results are reported as odds ratios with 95% confidence intervals (OR [95% CI]). Throughout, p-values below 0.05 are considered statistically significant.

## 3 Results

### 3.1 Study participants characteristics

Patients more frequently suffered from coronary artery disease (48%), than from cerebrovascular disease (5%), or peripheral artery disease (1.7%). Compared with healthy volunteers, FH, PCAD, but not AVS patients, exhibited higher levels of triglycerides, total, and LDL-cholesterol but lower levels of HDL-cholesterol (Fig. 1-**Panel A**). Plasma Lp(a) concentrations in the patient cohort were significantly higher than those of healthy subjects, with respective median Lp(a) of 70 and 30 nmol/L (p < 0.001). Similar to LDL-cholesterol, Lp(a) plasma concentrations were maximal in the FH subgroup at 89 (28–194) nmol/L. Kernel density analysis demonstrated a markedly right-skewed distribution of Lp(a) concentrations, with patients exhibiting a broader distribution and a pronounced tail toward higher levels (Fig. 1-**Panel B**). Their smaller and larger apo(a) isoforms contained on average 29 (25–32) and 34 (31–36) KIV domains, respectively. As anticipated [1], there was a significant negative correlation (r = −0.262; p < 0.001) between the concentration of Lp(a) and the size of the smaller isoform but not with the size of the larger isoform (r = −0.117; p = 0.179).

**Fig. 1:**
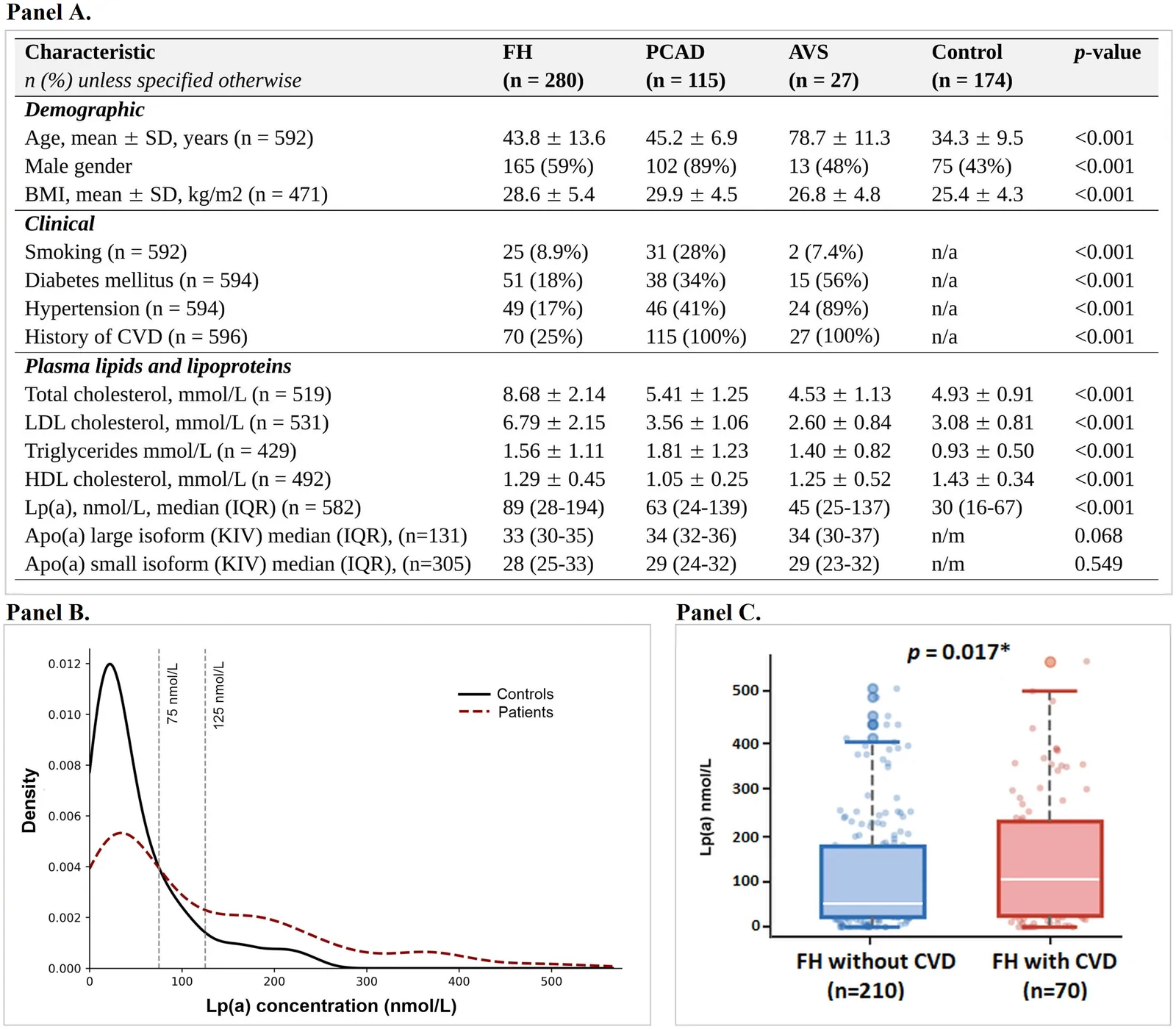
**Panel A.** Demographic, clinical, and plasma lipid parameters of study participants. Cardiovascular disease is defined by the presence of either coronary artery disease, cerebrovascular disease, or peripheral artery disease. Abbreviations: FH, familial hypercholesterolemia; PCAD, premature coronary artery disease; AVS, aortic valve stenosis; n/m, not measured; n/a, not applicable (by design, the controls were selected based on the absence of risk factors or co-morbidities). **Panel B.** Kernel density distribution of Lp(a) levels in healthy controls and patients. Dashed vertical lines indicate clinically relevant thresholds. **Panel C.** Plasma concentrations of Lp(a) in FH patients with and without CVD. Statistical significance was tested using the Mann–Whitney U test.

### 3.2 Association between Lp(a) and CVD risk

Given that all PCAD and AVS patients but none of the controls had suffered a cardiovascular event, we evaluated whether Lp(a) was a significant predictor of CVD only in the FH patient subgroup (n = 280). FH patients with a history of CVD had significantly higher Lp(a) concentrations compared to those without (p = 0.017) (Fig. 1-**Panel C)**. With CVD status set as the dependent variable and Lp(a) as the explanatory variable, multivariable logistic regression analyses showed that higher Lp(a) concentrations were independently associated with increased odds of CVD. Each 1 nmol/L increase in Lp(a) levels was associated with a 0.6% increase in CVD risk (OR: 1.006, 95% CI: 1.002–1.010; p = 0.002). Likewise, FH patients with Lp(a) concentrations ≥75 nmol/L were at significantly higher risk of CVD than those with Lp(a) concentrations <75 nmol/L (OR: 3.58, 95% CI: 1.38–9.33; p = 0.009). Similarly, Lp(a) concentrations ≥125 nmol/L were strongly associated with the risk of CVD (OR: 4.54; 95% CI: 1.63–12.65; p = 0.004).

## 4 Discussion

This study is the first to characterize Lp(a) levels, apo(a) isoform size, and their association with CVD in Oman. Lp(a) levels in healthy Omanis (30 nmol/L) were comparable to those of South Asians from the UK Biobank (31 nmol/L) [4]. In line with the established role of Lp(a) as a key driver of the early onset of atherosclerotic and valvular diseases [1], Lp(a) concentrations were higher in PCAD and AVS patients than in healthy controls. FH patients presented with even higher Lp(a) levels, reflecting the fact that high Lp(a) concentrations increase the likelihood for a patient to be clinically diagnosed as FH, and/or that elevated Lp(a) in FH may result from an impaired clearance of Lp(a) by the LDL receptor [5].

We uncovered that Lp(a) levels independently, significantly, and linearly increased the likelihood for a FH patient to suffer a major cardiovascular event. This highlights the additive detrimental effects of concomitantly elevated plasma Lp(a) and LDL cholesterol reported elsewhere [5], and suggests that lowering LDL cholesterol pharmacologically will not offset the specific cardiovascular risk stemming from elevated Lp(a) in FH.

To our surprise, we observed the coexistence of elevated Lp(a) plasma levels despite the expression of large apo(a) isoforms (≥23KIV domains) in a vast proportion of our patients. Unfortunately, we have not been able to determine the size of apo(a) isoforms in the healthy Omani group, which somewhat limits the scope of this observation. However, this finding contrasts with the well-established predominance of small apo(a) isoform carriers among individuals with elevated Lp(a) [1], and points toward an increased presence of genetic polymorphisms within or outside the *LPA* locus independent of the number of KIV repeats in this population. The presence of non-genetic factors that modulate Lp(a) production or clearance in a population-specific manner could also be at the origin of our observation. Indeed, circulating PCSK9 was found higher and correlated positively with the presence of Lp(a) particles carrying large apo(a) isoforms in African Americans but not Caucasians [6].

Our study is the first to characterize Lp(a) distribution in the Omani population. It provides key insights into the ethnic variability of Lp(a) and apo(a) isoforms in this specific population, paving the way for future research aimed at identifying Lp(a) increasing variants beyond apo(a) size polymorphism. Our observations advocate for a comprehensive assessment of Lp(a)-associated CVD risk in primary and secondary prevention Omani patients’ populations to define ethnically adequate therapeutic thresholds and optimal clinical management strategies for Oman and the Gulf region.

## Author agreement statement

We the undersigned declare that this manuscript is original, has not been published before and is not currently being considered for publication elsewhere.

We confirm that the manuscript has been read and approved by all named authors and that there are no other persons who satisfied the criteria for authorship but are not listed. We further confirm that the order of authors listed in the manuscript has been approved by all of us.

We understand that the Corresponding Author is the sole contact for the Editorial process. He/she is responsible for communicating with the other authors about progress, submissions of revisions and final approval of proofs.

## Funding support

This work was supported by Sultan Qaboos University Internal Grants (Grant No. IG/DVC/MRC/20/01), the Ministry of Higher Education, Research and Innovation, Oman, through the Block Funding Program (Grant No. RC/GRG-MED/BIOC/24/02), and the PerMedFH program grant LCF/PR/HP23/52330032 from La Caixa Foundation (Barcelona, Spain), awarded to GCL.

## CRediT authorship contribution statement

**Amani Al Khaifi:** Formal analysis, Visualization, Writing – original draft, Writing – review & editing. **Khalid Al Waili:** Investigation, Validation, Writing – review & editing. **Balaqis Al Shaibani:** Investigation, Validation, Writing – review & editing. **Hilal Al Sabti:** Investigation, Visualization, Writing – review & editing. **Adil Al Riyami:** Investigation, Validation, Writing – review & editing. **Manal Al-Kindi:** Investigation, Validation, Writing – review & editing. **Fahad Zadjali:** Investigation, Validation, Writing – review & editing. **Khalid Al-Saidi:** Investigation, Validation, Writing – review & editing. **Fahad Al Kindi:** Investigation, Validation, Writing – review & editing. **Hatim Al Lawati:** Investigation, Validation, Writing – review & editing. **Khamis Al Hashmi:** Investigation, Validation, Writing – review & editing. **Abrar Al Zadjali:** Investigation. **Razan Al Zadjali:** Investigation. **Sana Al Hasari:** Investigation. **Romuald Techer:** Investigation. **Kevin Chemello:** Investigation, Validation, Writing – review & editing. **Mirdavron Mukaddirov:** Validation, Writing – review & editing. **Koukab Al Farsi:** Investigation, Validation, Writing – review & editing. **Hassan Al Riyami:** Investigation. **Suaad Al Mukhaini:** Investigation. **Hala Al Badi:** Investigation. **Mohsin Al-Saleh:** Investigation, Validation, Writing – review & editing. **Eman R. Abdelmonaem:** Investigation. **Ibrahim Al-Zakwani:** Formal analysis, Validation, Writing – review & editing. **Gilles C. Lambert:** Conceptualization, Funding acquisition, Supervision, Validation, Writing – original draft, Writing – review & editing. **Khalid Al-Rasadi:** Conceptualization, Funding acquisition, Project administration, Supervision, Writing – review & editing.

## Declaration of competing interest

None of the authors has any financial interest or personal relationship which may be considered as potential competing interest to disclose.
